# The lasting influence of LSD1 in the blood

**DOI:** 10.7554/eLife.00963

**Published:** 2013-06-18

**Authors:** Sharon YR Dent, Joya Chandra

**Affiliations:** Department of Molecular Carcinogenesis, MD Anderson Cancer Center, Texas, United Statessroth@mdanderson.org; Department of Pediatrics Research and the Department of Molecular Carcinogenesis, MD Anderson Cancer Center, Texas, United Statesjchandra@mdanderson.org

**Keywords:** histone demethylase, Lsd1, KDM1, hematopoietic stem cells, granulocyte, enhancer, Mouse

## Abstract

An enzyme called LSD1 that controls the development of blood cells by manipulating gene expression in progenitor cells could be a therapeutic target for leukemia.

**Related research article** Kerenyi MA, Shao Z, Hsu Y-J, Guo G, Luc S, O’Brien K, Fujiwara Y, Peng C, Nguyen M, Orkin SH. 2013. Histone demethylase Lsd1 represses hematopoietic stem and progenitor cell signatures during blood cell maturation. *eLife* **2**:e00633. doi: 10.7554/eLife.00633

**Image** Mice that lack LSD1 (right) have fewer blood cells than control mice (left)

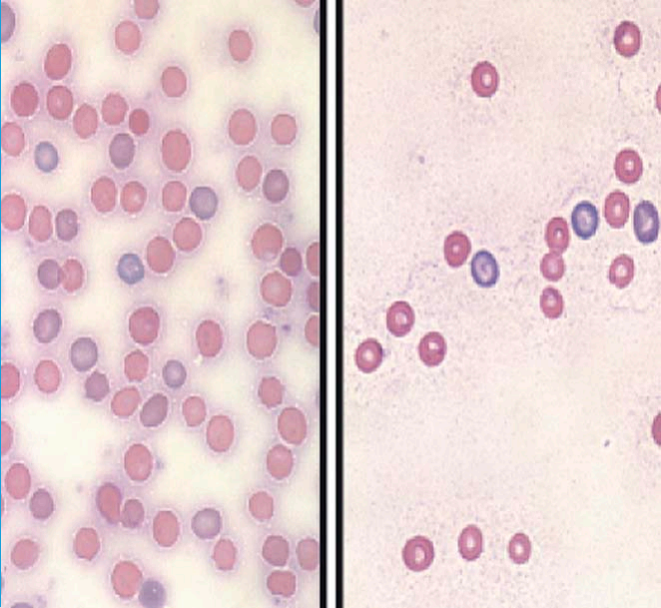

Inside the nucleus, DNA is tightly packaged around scaffold proteins called histones to form chromatin. Histones control how easy it is for proteins—notably those involved in transcribing the DNA sequence into messenger RNA—to access the DNA, so they have a critical role in regulating gene expression. Lysine residues within histones, particularly histones H3 and H4, are susceptible to many types of modification, including the addition of acetyl, methyl or ubiquitin groups. These modifications, often referred to as histone ‘marks’, influence how compact the chromatin is and help to recruit protein complexes that activate or repress transcription. Although knockouts of histone-modifying enzymes are often lethal in mice, gene deletions that are restricted to specific tissues or time periods provide valuable insights into how these enzymes choreograph cell fate. Now, in *eLife*, Stuart Orkin and colleagues at the Boston Children’s Hospital and the Dana Farber Cancer Institute reveal how a histone-modifying enzyme called lysine specific demethylase 1 (LSD1) affects the development of blood cells.

LSD1, which removes methyl groups from lysine 4 on histone H3 (H3K4), was the first histone demethylase to be discovered (Shi et al. 2004). H3K4 is methylated by enzymes from the MML (mixed lineage leukemia) family of methyltransferases, which are known to be important for the development of blood (Figure 1A) and are often altered in leukemias (Deshpande et al. 2012). Whether LSD1 inhibition might affect the development of blood cell progenitors has been explored in only one other study: last year researchers from University Hospital Essen used RNA-mediated gene silencing in mice to show that LSD1 depletion disrupts the formation of granulocytes (a type of white blood cell) and red blood cells, leading to acute anemia and a reduced number of platelets (Sprussel et al. 2012). While powerful, such knockdown models struggle to identify the role of LSD1 in the development of individual blood lineages, as they do not result in a complete loss of LSD1 expression. In the current study, Orkin and colleagues—including Marc Kerenyi as first author—overcame these limitations to reveal the functions of LSD1 in the formation of mature blood cells (hematopoiesis).

**Figure 1.**
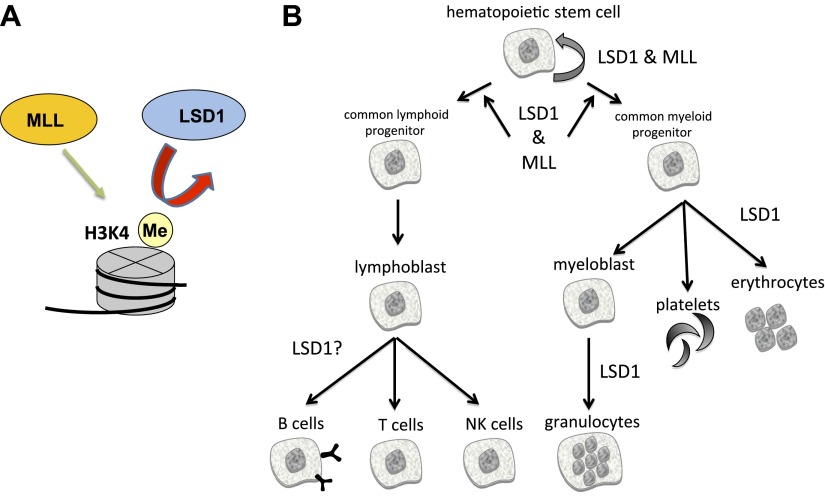
The enzymes LSD1 and MLL are both essential for the formation of blood cells (hematopoiesis), even though they have opposing roles in histone methylation. (**A**) MLL adds methyl groups (Me) to lysine 4 on histone 3 (H3K4), whereas LSD1 removes methyl groups from methylated H3K4. (**B**) Hematopoietic stem cells are pluripotent cells that give rise to myeloid and lymphoid progenitor cells, which in turn give rise to all lineages of blood cells. Conditional knockout mouse models reveal that both MLL and LSD1 are required for the maintenance of hematopoietic stem cells, and for the maintenance of myeloid and lymphoid progenitor cells. This is likely to be via shared roles in regulating the expression of Hox genes (not shown). LSD1 is also required for the production of erythrocytes (red blood cells) and granulocytes, and might be involved in the formation of B cells, T cells and NK cells.

All blood cells are derived from pluripotent cells called hematopoietic stem cells, which reside in bone marrow (Figure 1B). These give rise to two types of progenitor cells: lymphoid progenitors, from which immune cells called lymphocytes are derived; and myeloid progenitors, which give rise to all other types of blood cells. Kerenyi et al. used Cre-recombinase technology to delete two exons from Lsd1 that encode elements essential for catalysis. By systematically inactivating Lsd1 in different blood cell lineages, they identified roles for LSD1 in virtually all blood cell types. Collectively, their experiments revealed that the loss of LSD1 led to profound deleterious effects in both early and late hematopoiesis.

LSD1 has previously been implicated in stem cell pluripotency (Adamo et al. 2011), both in embryonic stem cells and in cancer stem cells. In the current study, bone marrow that contained LSD1 deficient cells was unable to replenish supplies of myeloid and lymphoid progenitors when transplanted into recipient mice. This indicates that loss of LSD1 reduced the ability of hematopoietic stem cells to replicate themselves. Altogether, these findings reveal that LSD1 is centrally important to several aspects of normal blood development, both in embryos and adults.

To determine how LSD1 regulates blood cell maturation, Kerenyi et al. examined gene expression in LSD1 deficient cells, and used a technique called ChIP-Seq—which maps interactions between DNA and proteins—to identify the DNA sequences to which Lsd1 binds in normal cells. Their data showed that many of the genes targeted by LSD1 are found in stem/progenitor cells. The data also revealed that gene expression networks involving specific Hox genes, which help to control embryonic development, are upregulated in the absence of LSD1. This provides a molecular explanation for the observed impairment of both early and late hematopoiesis in the absence of LSD1. Surprisingly, the data showed little association of LSD1 with known promoter regions or with patterns of H3K4 methylation that are hallmarks of promoters. Instead, LSD1 binding was found to be enriched at certain enhancer regions—DNA sequences remote from the transcription start site that bind proteins that upregulate transcription. These results indicate that LSD1 limits expression of Hox genes by inactivating enhancers, and dovetail neatly with previous reports that LSD1 ‘decommissions’ enhancers during the differentiation of embryonic stem cells (Whyte et al. 2012).

The phenotypes caused by LSD1 knockout—including impaired blood cell formation and aberrant Hox gene expression—are reminiscent of those caused by mutation of some MLL family methyltransferases (Figure 1B) (Hess, 2004). Why would mutation of enzymes that have opposing functions (removing versus adding methyl groups) have similar phenotypes? At least part of the answer lies in the discovery that chromosomal translocations can give rise to MLL fusion proteins that contain a ‘super elongation complex’. This increases elongation of Hox gene transcripts, thereby increasing Hox expression just as loss of LSD1 does. Also, MLL proteins and LSD1 both function as components of multi-protein complexes, so developmental issues arising from mutations in these factors could reflect effects on a third party conspirator that interacts with both complexes. Furthermore, non-histone proteins can be substrates for both methyltransferases and demethylases. LSD1, for example, is documented to demethylate several non-histone proteins (Nicholson and Chen, 2009), pointing to additional potential mediators of the observed effects on hematopoiesis.

Several tumor types show heightened expression of LSD1 (Amente et al. 2013), suggesting that it may function as an oncogene. Studies in acute myeloid leukemia models further support this idea. Two independent groups have reported that LSD1 inhibition has anti-leukemic effects in cell lines and in mouse models of human leukemia (Harris et al. 2012; Schenk et al. 2012). In light of such findings, pharmaceutical companies are rapidly developing LSD1 inhibitors for therapeutic uses. However, whether inhibition of LSD1 will hinder development of non-leukemic cells, which would limit its potential as a therapeutic target, has been a lingering question, particularly in light of data demonstrating that mice harboring germline deletion of LSD1 die early in development (Foster et al. 2010). The findings of Kerenyi et al. raise additional questions about the feasibility of LSD1 as a drug target, as loss of LSD1 affects multiple aspects of hematopoiesis. On the other hand, drugs such as tranylcypromine, an LSD1 inhibitor that is used in the treatment of neurological disorders (Lee et al. 2006), do not cause severe toxicities in mice or humans. Clearly, preclinical and clinical evaluation of any newly developed LSD1 inhibitors must address potential adverse effects on the blood, which are a common side effect of many cancer therapies.
